# eLighting up the future

**DOI:** 10.1038/s41377-021-00555-0

**Published:** 2021-06-08

**Authors:** Ling Lu

**Affiliations:** Institute of Physics, Chinese Academy of Sciences, Beijing, China

**Keywords:** Optics and photonics, Physics

## Abstract

What will be the cutting-edge photonics research in the coming decade? Prof. Chen and Segev share their perspective by highlighting quantum, topological, and AI photonics on *eLight*.

For graduate students looking for thesis topics, professionals keeping up with the latest directions, or funding managers identifying the next promising projects in photonics, a timely and well-crafted analysis^1^ is presented in the opening issue of *eLight*. This perspective is written by two renowned scientists, Prof. Zhigang Chen and Moti Segev, who highlighted three major frontiers in photonics that will most likely undergo exciting growth in the next decade (2021–2030): integrated quantum photonics, topological photonics, and artificial-intelligence (AI) photonics.

With the significant amount of funding from state governments as well as big companies, it is clear that quantum science will continue to take center stage in the near future. The holy grail of quantum technology is the long-term goal of making a fault-tolerant universal quantum computer. Among various approaches under keen investigations, Chen and Segev put their bets on integrated quantum optics. The main argument is the low coupling between photons and the environments, allowing room-temperature operations with the lowest error rates compared to other scalable platforms based on matter-qubits. At least two startup companies, PsiQuantum (https://psiquantum.com) and Xanadu (https://xanadu.ai), are already on their way to produce quantum photonic chips.

Topological photonics is a uniquely new field whose underlying principles are not covered in existing electromagnetics or optics textbooks. (Check out its latest developments on the special issue of *Light: Science & Applications*^2^). The field was initiated from a theory of the 2016 Nobel-laureate Duncan Haldane, who proposed the perfect (one-way) waveguiding mechanism at the edge of two-dimensional magnetic photonic crystals. Although time-reversal breaking is the necessary condition for complete back-scattering immunity, researchers have to relax this condition due to the weak magneto-optical effect at optical wavelengths. Although this comes with the penalty of reopening the back-scattering channels and conditional robustness, these non-magnetic topological waveguides are still stabler than their traditional counterparts. Chen and Segev emphasized the idea of using these edge channels to couple VCSEL arrays, addressing the long-standing issue of super-mode instability in phase-locked laser arrays that are intrinsically non-Hermitian and nonlinear devices.

The sweeping success of two projects from Google’s DeepMind has reshaped our perception of AI, with Alpha-Go crashing the best go-players and Alpha-Fold predicting protein structures at unprecedented accuracy. We might be seeing the dawn of the AI age, as AI algorithms and products are penetrating our daily lives at an increasing rate, ranging from speech recognition, medical diagnosis to robotics. On one hand, AI can be used to predict photonic designs without solving Maxwell’s equations, on the other hand, photonic hardware can accelerate AI by performing machine-learning computations using photons with much higher throughput and lower energy consumption. A few startups are already building such optical AI-accelerators, including Lightelligence (https://lightelligence.ai), Lightmatter (https://lightmatter.co), Luminous (https://luminous.co) among others.

**Fig. 1:**
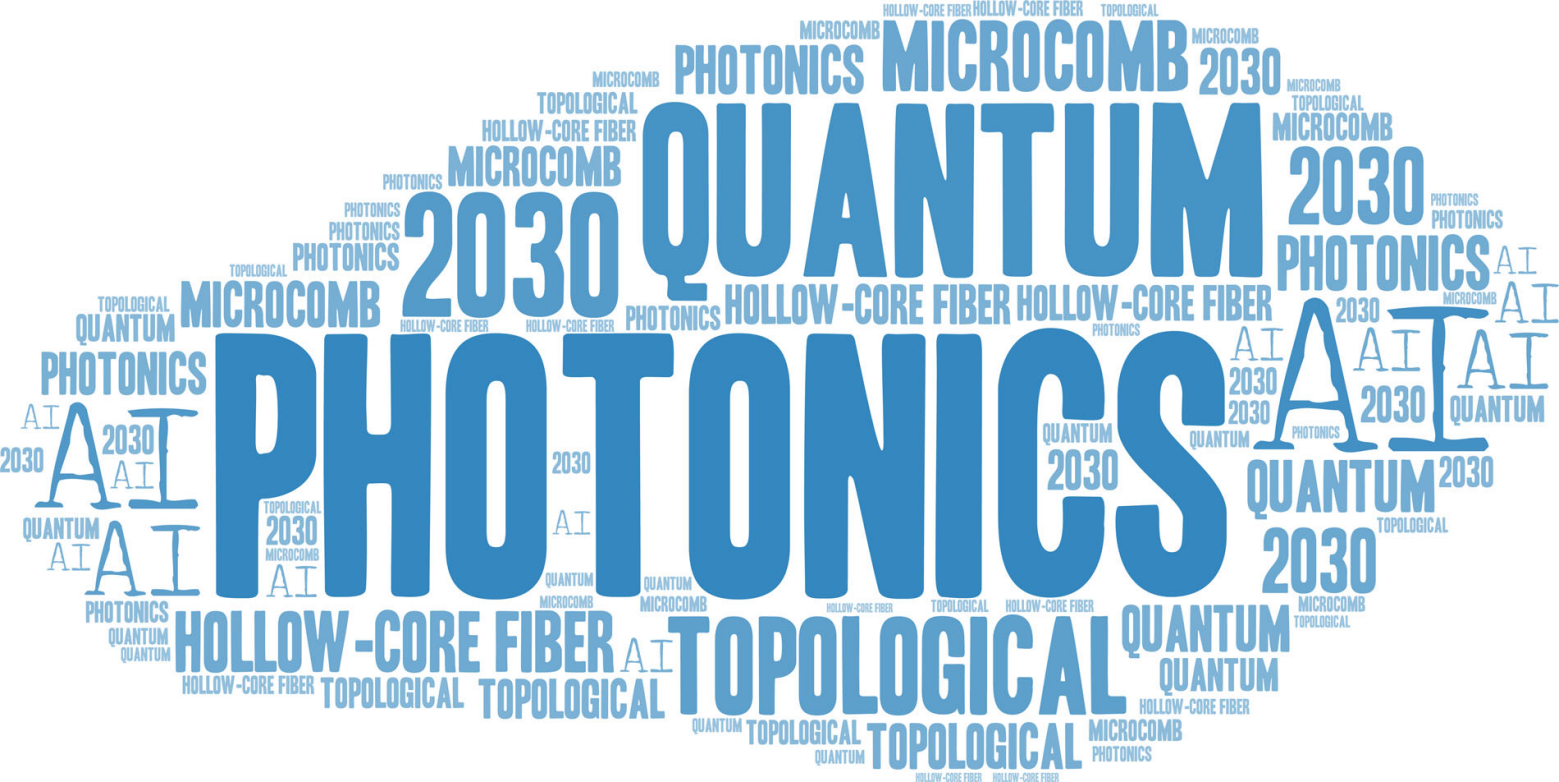
Selected keywords for active photonic research in the next decade (2021–2030). Figure made on WordArt.com

Other than the aforementioned three areas featured by Chen and Segev, there are certainly many other exciting directions that are growing tremendously. Here in Fig. 1, I just add a couple. One is the microcomb—optical frequency combs in micro-resonators, an emerging disruptive innovation that integrates the Nobel-prize (2005) winning technology on a turnkey chip^3^. The other is the hollow-core fiber^4^, whose propagation loss is expected to break the 0.14 dB/km record of silica fiber! Of course, what we anticipate the most are those ground-breaking discoveries that no one expects.
